# Prenatal androgen exposure predicts sexuality disorders: insights from anthropometric measurements and questionnaires

**DOI:** 10.3389/fendo.2025.1546385

**Published:** 2025-03-13

**Authors:** Adrian Bartoszek, Magdalena Sawic, Karol Pierzchała, Aleksandra Kudrycka, Piotr Białasiewicz, Wojciech Kuczyński

**Affiliations:** ^1^ Department of Bioanalytics, Medical University of Lublin, Lublin, Poland; ^2^ Independent Unit of Spectroscopy and Chemical Imaging, Medical University of Lublin, Lublin, Poland; ^3^ Department of Sleep Medicine and Metabolic Disorders, Medical University of Lodz, Lodz, Poland

**Keywords:** digit ratio, sexuality disorders, 2D:4D, PAE, finger length

## Abstract

**Background:**

Sexual activity has been linked to various physical and psychological benefits, yet national surveys indicate a decrease in sexual engagement among American adults from the late 1990s to the early 2010s. The 2D:4D ratio, representing the relative lengths of the second and fourth digits, is commonly used as a biomarker for prenatal androgen exposure (PAE). This ratio may offer insights into the hormonal environment during fetal development, which could impact sexual attitudes and mental well-being. This study aimed to explore the associations between PAE, inferred via 2D:4D ratio, and various psychosocial factors, including sexual attitudes, mental health, and self-reported sexual satisfaction.

**Methods:**

A cohort of male and female participants was assessed for 2D:4D ratios on both hands. Questionnaires captured a range of psychosocial and sexual measures, including the Arizona Sexual Experiences Scale (ASEX), the Sexual Satisfaction Questionnaire (SSI), the Sapiosexual Questionnaire (SapioQ), the Kinsey Scale for sexual orientation, and tools assessing mental health and quality of life (SF-12, PHQ-9, GAD-7, MDQ, PSQI). Statistical analyses were conducted to identify correlations between PAE, mental health, and sexuality, with gender differences considered.

**Results:**

Women reported higher ASEX and SSI scores but lower SF-12 mental and physical health scores than men, consistent with smaller 2D:4D effect sizes reported in previous research. Overall, PAE did not correlate strongly with general mental health or sexual satisfaction. However, high PAE was associated with a greater openness to casual relationships, particularly among women, while low-PAE individuals prioritized intelligence over physical traits in partner preferences.

**Conclusions:**

These findings suggest that PAE, as measured by the 2D:4D ratio, may be associated with certain adult psychosocial traits. Although correlations were weak, this study contributes to understanding the subtle role of PAE in shaping sexual attitudes and mental health, highlighting the need for further research in more diverse populations.

## 1 Introduction

Sexual function is a critical component of overall quality of life, with androgens playing a key role in its regulation for both men and women. Testosterone, often regarded as a male hormone, is vital for both sexes. It influences numerous physiological processes, including mood, motivation, muscle strength, cognitive performance, and memory formation (1). In a seminal study, Manning et al. proposed that a lower 2D:4D ratio, defined as the length ratio of the second to the fourth digit, indicates elevated prenatal testosterone exposure in humans (2). This parameter, also known as the digit ratio (DR), is proposed to reflect sexual satisfaction. A higher exposure to testosterone and a lower exposure to estrogen hormones are associated with a lower 2D:4D ratio (3). The DR remains relatively stable from birth or early infancy and shows no significant correlation with adult sex hormone levels. Rather than reflecting androgen concentrations, it appears to be associated with androgen sensitivity. Research has linked variations in DR to a wide range of behavioral traits (assertiveness, aggression, and anxiety), physiological characteristics (obesity, handedness, and sperm count), and various health outcomes (4). Moreover, several diseases from various medical branches have been correlated to the DR. To comprehend the neurobiological processes underlying sexual disorders fully, it is essential to understand the role of sex hormones during the prenatal period and how these hormones interact with sexual predispositions. The single finger lengths may also be a marker of the exposure. Literature emphasizes the importance of raw data (like single finger lengths) to improve accuracy and ensure the reliability of findings across different methodologies (5). Analyzing single finger lengths allows researchers to explore whether observed sex differences in traits are driven by absolute finger lengths, the 2D:4D ratio, or both. This is particularly relevant when assessing the robustness of sex-dimorphic traits.

The Organizational and Activational Hypothesis posits that the effects of sex hormones on behavior are twofold: early in development, they organize the brain and body in a sex-specific manner, and later in life, hormonal changes, particularly during puberty, activate these structures (6). This theory suggests that both prenatal hormone exposure and adult hormone levels contribute to sexual differentiation and related behaviors. Some evidence supports the Organizational and Activational Hypothesis concerning human sexuality; however, these studies have notable limitations. For instance, a significant majority (93%) of 46XY individuals with androgen insensitivity syndrome (AIS) exhibit androphilia, meaning they are primarily attracted to men (7). Moreover, several researchers explored the association between 2D:4D and sexual orientation (8). To our best knowledge, Breedlove et al. were the leading team to find the correlation of DR in anfrophilic females and no association in men (9). Another interesting finding was a meta-analysis from Siegmann et al. (10). A meta-analysis, including 17 samples and over 3,600 participants, reinforced these findings, showing a significant difference in 2D:4D between male to female (MtF) transgender identity individuals and male controls. These findings suggest that variations in 2D:4D, particularly in MtF individuals, reflect the influence of PAE on gender identity, adding evidence to the role of early hormone environment in shaping sexual and gender preferences.

In this study, sexual function was assessed through the Arizona Sexual Experiences Scale (ASEX), a brief, five-item measure that provides self-reported insights into key areas of sexual activity, including sexual desire, arousal, erectile function, orgasmic ease, and satisfaction. To deepen the understanding of sexual health, additional assessments were employed, such as the Sexual Satisfaction Questionnaire (SSQ) across domains of Closeness, Petting, and Sex, along with the Sapiosexual Questionnaire (SapioQ) and the Sexual Satisfaction Index (SSI), offering a comprehensive view of individual sexual function. The primary aim was to explore potential correlations between prenatal androgen exposure (PAE), indicated as the 2D:4D digit ratio (DR), and sexual disorders, using a broad range of assessments across sexuality, depression, anxiety, and sleep to capture the multifaceted aspects of sexual health and mental well-being.

## 2 Materials and methods

### 2.1 Study design

This study is part of the research project “Understanding the associations of PAE on sleep physiology, circadian proteins, anthropometric parameters, hormonal factors, quality of life and sex among healthy young adults – BOAT international, multicenter study (2021). This project involved collaboration with researchers from New Zealand, UK, and Japan. However, due to the challenges posed by the COVID-19 pandemic, data collection by the international teams was not feasible. Despite these setbacks, the Polish team successfully collected data, leading to the publication of the research protocol (8). Surveys were conducted from March 2020 to March 2022. The research protocol has been detailed in previous publications (8). Inclusion criteria consist of a full understanding of the study rules by the participant, confirmed by written informed consent to participate in the study and an age range of 18 to 30 years. Exclusion criteria consist of a diagnosis of chronic, hormonal, and mental health conditions, pregnancy and lactation, taking long-term medicines including hormonal contraception, injuries to the fingers of the upper limbs, deformation of the fingers of the upper limbs, diseases leading to deformation of the fingers of the upper limbs, and lack of consent or inability to follow recommendations related to participation in the study. Data was collected from 720 participants, with 290 completing all questionnaires. These respondents were subsequently invited to participate in a follow-up measurement session, with 138 attending and undergoing anthropometric measurements.

### 2.2 Measurements tools

Participants completed various questionaries, including:

- Demographic: Collected information on ethnicity, sex, and gender.
- Arizona Sexual Experiences Scale (ASEX): Validated five-item questionnaire designed to assess various aspects of sexual functioning, including sexual drive, arousal, penile erection or vaginal lubrication, ability to reach orgasm, and satisfaction with orgasm over the past week (11). The ASEX’s straightforward format allows for both self-reporting and clinician administration, making it a versatile tool for assessing sexual health.
- Sapiosexual Questionnaire (SapioQ) designed to assess individuals’ attraction to intelligence as a primary factor in their romantic and sexual preferences. This concept aligns with findings that suggest intelligence can significantly influence mate desirability, with research indicating that characteristics such as IQ can peak in desirability but may also exhibit threshold effects, where exceedingly high intelligence could detract from attractiveness (12).
- Sexual Satisfaction Questionnaire is a research tool designed to assess the level of sexual satisfaction within a relationship, with higher scores reflecting greater satisfaction. It consists of ten questions grouped into three main categories: closeness, petting, and sexual relations (13).
- Sexual Satisfaction Index (SSI) is a tool designed to measure an individual’s level of sexual satisfaction, with higher scores indicating a greater level of satisfaction. It serves not only to evaluate sexual satisfaction but also to explore its connection to overall quality of life, self-esteem, and relationship quality, making it valuable for both clinical assessments and research purposes (14).
- Kinsey Scale is a tool used to classify an individual’s sexual orientation on a continuum from exclusively heterosexual (0) to exclusively homosexual (6). This scale recognizes that sexual orientation is not binary but exists along a spectrum, allowing for a more nuanced understanding of human sexuality (15).
- Generalized Anxiety Disorder Screener (GAD-7) is a widely used seven-item screening tool for generalized anxiety disorder, quantifying the severity of anxiety symptoms experienced over the past two weeks (16).
- Mood Disorder Questionnaire (MDQ) is designed to screen for the presence of mood disorders, including bipolar disorder. It asks about a lifetime history of mood swings, diagnosis, and treatment, providing insights into the mental health status of participants (17).
- Patient Health Questionnaire-9 (PHQ-9) is a nine-item self-assessment tool for evaluating the severity of depressive symptoms in both clinical and research settings. It consists of nine items based on DSM-IV criteria for major depression, asking respondents to indicate the frequency of symptoms experienced over the past two weeks (18).
- Pittsburgh Sleep Quality Index (PSQI) assess sleep quality and disturbances over one month, evaluating seven domains: sleep latency, duration, efficiency, disturbances, medication use, daytime dysfunction, and overall sleep quality. This yields a comprehensive overview of an individual’s sleep patterns (19).
- Short Form Survey (SF-12) is a concise self-report questionnaire designed to assess health-related quality of life across two main dimensions: physical (PCS) and mental (MCS) health. Derived from the longer SF-36, the SF-12 retains essential items that allow it to effectively evaluate an individual’s perceived health status while being shorter and quicker to administer (20).

The direct measurements of the second and fourth fingers on both hands were performed by qualified staff using a sliding caliper (Vernier caliper) with an accuracy of 0.001 m. Based on the values of the fingers’ length, the 2D:4D index was calculated as a quotient of the length of the second digit and the fourth digit (mm). Measurements were performed on the palmar side of the hand using anthropometric points lying on the digit axis: pseudophalangion—a point in the finger metacarpophalangeal crease, dactylion—the most distal point on the fingertip. A 2D:4D ratio > 1 was considered as a low PAE, while a ratio <1 was considered as a high PAE. Handedness was defined via self-report.

### 2.3 Data collection

All participants were aware of the study conditions and gave informed consent to participate. Confidentiality and anonymity were maintained, and no data that could help identify a responder were collected. The study was conducted in accordance with the amended Declaration of Helsinki, and the Ethics Committee of the Medical University of Lodz approved the study protocol (RNN/394/19/KE); 2020/04/X/NZ4/00564.

### 2.4 Statistical analysis

Statistical analysis was performed using STATISTICA 13.1 (TIBCO, Palo Alto, Santa Clara, CA, USA). A level of 5% was used as a significance threshold for all the results unless stated otherwise. In the case of multiple testing, the Bonferroni correction was used. No data obtained had normal distribution (Shapiro–Wilk’s test, p < 0.05). The relationship between the independent subgroups was assessed using the Mann-Whitney U test, where the effect size was reported as *r* and interpreted as weak (*r* < 0.3), moderate (0.3 ≤ *r* < 0.5), or strong (*r* ≥ 0.5). In the tables, effect size was indicated using lowercase letters next to numerical values: *w* (weak), *m* (moderate), and *s* (strong). The relationship between continuous variables was assessed using the Spearman correlation coefficient. The false discovery rate (FDR) correction was applied when the Spearman correlation was used. If the *p*-value was not significant after FDR correction, an asterisk (*) was placed next to the corresponding value in the table.

## 3 Results

### 3.1 Study group

The study included 138 Polish students with an average age of 22.28 ± 1.99 years with no medical conditions and no previous hand-injury. Among them, 61.6% were women. 94.2% of the respondents were right-handed. Anthropometric measurements and PAE, considering the sex of the respondents, are presented in **Table 1**. Men have larger 2nd and 4th fingers, while women have a significantly larger 2D:4D ratio (**Table 1**). For subsequent analyses, measurements taken from the dominant hand were used.

**Table 1:** Anthropometric measurements.

Anthropometric measurements	Mean	Gender	
		Female (61.59%)	Male (38.41%)
2D _L_	70.62 (4.72)	68.49 (3.98)	74.02 (3.72)
4D _L_	71.09(5.19)	68.78 (4.46)	74.79 (4.03)
2D:4D _L_	0.99 (0.03)	0.99 (0.03)	0.99 (0.03)
2D _R_	71.04 (4.60)	69.13 (4.22)	74.11 (3.23)
4D _R_	71.33 (5.09)	68.99 (4.50)	75.09 (3.49)
2D:4D _R_	0.99 (0.03)	1.00 (0.03)	0.99 (0.03)
2D:4D _mean_	0.99 (0.03)	1.00 (0.03)	0.99 (0.03)
2D *_handedness_*	71.04 (4.55)	69.12 (4.20)	74.13 (3.22)
4D *_handedness_*	71.30 (5.07)	68.99 (4.48)	75.02 (3.53)
2D:4D *_handedness_*	0.99 (0.03)	1.00 (0.03)	0.99 (0.03)

In red bold-statistically significant differences. _L- left hand measurement; _R- right hand measurement; _handedness - dominant hand measurement. Data presented as mean (SD).

### 3.2 Relationship between anthropometric measurements, PAE, sexuality and mental disorders

**Table 2** presents the average scores for the questionnaires used in the study. Women have significantly higher ASEX and SapioQ scores than men but lower SSQ (Sex), SF-12 (MCS) and SF-12 scores. No other gender differences were observed (**Table 2**). PAE (measured on the dominant hand) does not differentiate any of the questionnaires used in the study, also when considering gender (**Table 2**).

**Table 2:** Mann-Whitney test results for 2D:4D and PAE, where PAE is assessed on the dominant hand.

	All	Female	Male	PAE		PAE			
						Male (50%)		Female (50%)	
				High (50%)	Low (50%)	High (60.38%)	Low (39.62%)	High (43.53%)	Low (56.47%)
ASEX	11.23 (4.27)	**12.36^m^** **(4.63)^###^**	**9.42^m^** **(2.8)^###^**	11.20 (4.49)	11.26 (4.07)	9.12 (2.49)	9.86 (3.24)	13.0 (5.06)	11.88 (4.27)
SSQ (Closeness)	18.49 (8.08)	18.81 (7.98)	17.98 (8.28)	19.03 (7.55)	17.96 (8.6)	17.09 (8.35)	19.33 (8.19)	20.7 (6.44)	17.35 (8.78)
SSQ (Petting)	6.01 (3.03)	5.74 (3.05)	6.45 (2.98)	6.06 (2.98)	5.97 (3.1)	6.19 (3.19)	6.86 (2.65)	5.95 (2.83)	5.58 (3.23)
SSQ (Sex)	6.13 (3.83)	**5.68^w^** **(3.76) ^#^**	**6.85^w^** **(3.86) ^#^**	6.46 (3.73)	5.8 (3.92)	7.19 (3.76)	6.33 (4.04)	5.84 (3.64)	5.56 (3.89)
SSQ	30.64 (13.84)	30.24 (13.72)	31.28 (14.15)	31.55 (13.06)	29.72 (14.63)	30.47 (14.6)	32.52 (13.71)	32.49 (11.69)	28.5 (14.98)
SapioQ	3.37 (0.60)	**3.49^w^** **(0.63)^##^**	**3.17^w^** **(0.5)^##^**	3.33 (0.57)	3.41 (0.64)	3.2 (0.54)	3.12 (0.43)	3.44 (0.58)	3.53 (0.67)
SSI	19.13 (9.90)	18.69 (10.31)	19.83 (9.25)	17.84 (10.85)	20.42 (8.74)	18.06 (10.76)	22.52 (5.5)	17.65 (11.07)	19.5 (9.73)
PHQ-9	7.12 (5.72)	7.39 (6.22)	6.68 (4.85)	7.30 (6.29)	6.93 (5.14)	8.32 (7.17)	6.67 (5.34)	6.12 (4.94)	7.52 (4.72)
GAD-7	4.98 (4.82)	5.26 (5.14)	4.53 (4.28)	5.06 (5.36)	4.9 (4.26)	5.97 (5.98)	4.71 (4.37)	4.0 (4.39)	5.33 (4.07)
MDQ	41.30 (31.24)	38.1 (31.24)	46.44 (30.84)	39.46 (32.26)	43.14 (30.32)	38.46 (32.53)	37.82 (30.56)	40.62 (32.41)	55.31 (26.6)
PSQI	6.94 (2.82)	7.02 (2.95)	6.81 (2.63)	7.35 (3.14)	6.54 (2.42)	7.65 (3.51)	6.54 (2.35)	7.0 (2.65)	6.52 (2.64)
SF-12 (PCS)	17.09 (1.98)	16.89 (2.06)	17.40 (1.82)	17.31 (2.03)	16.86 (1.92)	17.66 (1.82)	17.00 (1.79)	17.03 (2.18)	16.79 (1.99)
SF-12 (MCS)	18.27 (4.45)	**17.59^w^** **(4.32)^##^**	**19.36^w^** **(4.47)^##^**	18.38 (4.33)	18.16 (4.59)	19.44 (4.23)	19.24 (4.91)	17.46 (4.25)	17.69 (4.42)
SF-12	35.36 (5.91)	**34.48^w^** **(5.74)^#^**	**36.75^w^** **(2.63)^#^**	35.69 (5.81)	35.01 (6.03)	37.09 (5.38)	36.24 (6.35)	34.49 (5.97)	34.48 (5.88)

Significant differences are highlighted in red. Data are reported as mean (SD). Additionally, effect sizes were calculated using r, with interpretation thresholds of weak (w) and moderate (m). Significance levels are indicated as follows: ^#^ *p* < 0.05, ^##^ *p* < 0.01, ^###^ *p* < 0.001.

When taking into account the exact anthropometric measures taken on the dominant hand (**Table 3**), the initial analysis revealed statistically significant associations with the questionnaires. However, after applying the FDR correction, none of these correlations remained statistically significant (**Table 3**).

**Table 3:** Spearman Correlation between anthropometric measurements and scales regarding sexuality and mental disorders.

	2D			4D			2D:4D		
	All	Male	Female	All	Male	Female	All	Male	Female
ASEX	-0.14	0.03	0.05	-0.14	-0.04	0.10	0.02	0.10	-0.11
SSQ (Closeness)	-0.04	0.03	-0.04	0.03	0.05	0.09	-0.14	-0.03	-0.24*
SSQ (Petting)	0.15	0.05	0.13	0.19*	0.08	0.18	-0.11	-0.05	-0.11
SSQ (Sex)	0.08	0.01	-0.01	0.15	0.15	0.04	-0.17*	-0.19	-0.12
SSQ	0.03	0.04	0.00	0.10	0.09	0.10	-0.15	-0.08	-0.20
SapioQ	-0.20*	-0.06	-0.07	-0.22*	-0.10	-0.09	0.10	0.06	0.04
SSI	-0.02	0.09	-0.11	-0.09	-0.06	-0.17	0.13	0.21	0.11
PHQ-9	-0.18*	0.15	-0.29*	-0.14	0.11	-0.22*	-0.06	0.04	-0.13
GAD-7	-0.15	0.21	-0.27*	-0.12	0.20	-0.22*	-0.04	-0.01	-0.07
MDQ	0.07	0.26	-0.11	0.08	0.11	-0.05	-0.03	0.19	-0.13
PSQI	-0.10	0.18	-0.21	-0.04	0.29*	-0.15	-0.11	-0.17	-0.09
SF-12 (PCS)	0.07	0.03	-0.02	0.08	0.12	-0.03	-0.05	-0.13	0.03
SF-12 (MCS)	0.06	-0.03	-0.06	0.05	-0.08	-0.09	0.01	0.06	0.05
SF-12	0.07	-0.02	-0.05	0.06	-0.02	-0.07	-0.1	0.00	0.05

Measures were conducted on the dominant hand. The significant correlations have been bolded in red. After applying the FDR correction, if the p-value exceeded the significance threshold, an asterisk (*) was added next to the value to indicate the adjustment.

### 3.3 Relationship between sexuality and mental disorders

Then, questionnaires regarding sexuality were correlated with those assessing mental disorders (**Table 4**). In our study group, ASEX was negatively correlated with SF-12(MCS), but the correlations were weak. SSI was positively correlated with questionnaires regarding mental disorders (PHQ-9, GAD-7, MDQ) and negatively correlated with SF-12 and its subscales. The SapioQ questionnaire was negatively correlated with SF-12 and its subscales. In women, SSI was positively correlated with PHQ-9 and GAD-7 in both high and low participants, but MDQ was only correlated in the high PAE group. In men, SSI was also positively correlated in high PAE males with PHQ-9, GAD-7, and MDQ (**Table 4**).

**Table 4:** Spearman Correlation between scales regarding sexuality and mental disorders.

	Gender	PAE	PHQ-9	GAD-7	MDQ	PSQI	SF-12 PCS	SF-12 MCS	SF-12
ASEX	All		0.07	0.08	0.04	-0.03	-0.09	**-0.23**	-0.20*
SSQ (Closeness)			0.09	0.08	0.11	-0.05	-0.06	-0.03	-0.04
SSQ (Petting)			0.00	0.02	0.09	-0.05	-0.03	0.04	0.02
SSQ (Sex)			0.10	0.12	0.11	-0.02	-0.00	0.11	0.08
SSQ			0.11	0.11	0.11	-0.06	-0.06	0.01	-0.01
SapioQ			0.07	0.06	0.03	-0.02	**-0.22**	**-0.21**	**-0.23**
SSI			**0.57**	**0.58**	**0.49**	0.19*	**-0.23**	**-0.22**	**-0.23**
ASEX	F	high	0.11	0.10	0.03	0.08	-0.17	-0.27	-0.24
		low	0.08	0.04	0.17	0.05	0.02	-0.16	-0.11
SSQ (Closeness)		high	0.02	-0.15	0.12	-0.10	-0.06	-0.06	-0.05
		low	0.11	0.15	0.18	-0.10	-0.09	-0.08	-0.11
SSQ (Petting)		high	-0.03	-0.12	-0.08	0.00	-0.25	-0.16	-0.17
		low	0.03	0.07	0.26	-0.13	0.06	0.06	0.05
SSQ (Sex)		high	0.04	-0.07	-0.09	-0.05	-0.20	-0.05	-0.08
		low	0.04	0.11	0.19	-0.14	0.08	0.15	0.12
SSQ		high	0.03	-0.14	0.02	-0.10	-0.20	-0.09	-0.10
		low	0.09	0.16	0.17	-0.12	-0.06	-0.02	-0.06
SapioQ		high	0.07	0.15	0.17	-0.17	-0.16	-0.19	-0.17
		low	0.04	-0.04	0.05	0.05	-0.22	-0.06	-0.11
SSI		high	**0.64**	**0.58**	**0.65**	0.33*	-0.26	-0.31	-0.30
		low	**0.53**	**0.59**	0.37*	0.24	-0.29*	-0.22	-0.24
ASEX	M	low	-0.27	-0.14	-0.04	-0.15	0.04	-0.24	-0.18
		high	0.10	0.20	0.11	-0.43*	-0.24	-0.16	-0.21
SSQ (Closeness)		low	0.11	0.16	0.10	0.15	0.05	0.39	0.31
		high	0.11	0.10	0.04	-0.16	-0.05	-0.13	-0.12
SSQ (Petting)		low	-0.01	-0.03	0.01	0.06	0.00	0.18	0.15
		high	0.04	0.06	0.05	-0.04	-0.03	-0.13	-0.13
SSQ (Sex)		low	0.36	0.43	0.20	0.18	-0.03	0.17	0.10
		high	0.21	0.16	0.13	0.06	-0.01	0.01	0.03
SSQ		low	0.17	0.23	0.16	0.10	0.01	0.34	0.26
		high	0.17	0.14	0.10	-0.13	-0.05	-0.11	-0.11
SapioQ		low	0.12	0.20	0.11	0.16	-0.23	-0.43	-0.43*
		high	0.11	0.19	0.06	-0.16	-0.22	-0.24	-0.26
SSI		low	0.16	0.20	0.11	-0.01	-0.06	-0.06	-0.07
		high	**0.64**	**0.68**	**0.70**	0.03	-0.05	-0.18	-0.10

PAE measured on the dominant hand. The significant correlations have been bolded in red. After applying the FDR correction, if the p-value exceeded the significance threshold, an asterisk (*) was added next to the value to indicate the adjustment.

### 3.4 Relationship between PAE and sexual orientation

No differences in sexual orientation were observed in participants when taking into account PAE and Gender (**Table 5**).

**Table 5:** Kinsey Scale. PAE is measured on the dominant hand.

Kinsey Scale	PAE		PAE			
			Male		Female	
	High	Low	High	Low	High	Low
0 Exclusively heterosexual	34.1	31.9	29.4	32.9	41.5	30.2
1 Predominantly heterosexual. only incidentally homosexual	8.0	10.1	8.2	14.1	7.5	3.8
2 Predominantly heterosexual. but more than incidentally homosexual	2.2	2.9	2.4	4.7	1.9	0.0
3 Equally heterosexual and homosexual	2.2	1.4	1.2	2.4	3.8	0.0
4 Predominantly homosexual. but more than incidentally heterosexual	0.0	0.7	0.0	0.0	0.0	1.9
5 Predominantly homosexual. only incidentally heterosexual	2.2	0.0	0.0	0.0	5.7	0.0
6 Exclusively homosexual	0.0	1.4	0.0	0.0	0.0	3.8
x No socio-sexual contacts or reactions (asexual)	1.4	1.4	2.4	2.4	41.5	30.2

Data are presented as %.

### 3.5 Relationship between PAE and sexual behaviors

Individuals with high PAE were more likely to have engaged in a “friends with benefits” relationship (22%) compared to those with low PAE (15%), suggesting a greater openness to this type of relationship among those with higher PAE. Similarly, when considering the idea of a “one-night stand,” 30% of participants with high PAE reported having considered it, while only 22% of those with low PAE expressed similar thoughts. The same trend was observed in females but not males (**Table 6A**). Individuals with low PAE more often declared that intelligence and intriguing personalities attracted them to sexual partners, but less often it was physical attractiveness and self-confidence. No differences were observed when considering the gender (**Table 6B**).

**Table 6A:** Self-created questionnaires regarding sexuality.

Question	Answer	PAE		PAE			
				Female		Male	
		high	low	high	low	high	low
Have you ever had “one-night stand”?	Yes	11	13	5	12	40	25
	No	39	37	39	45	21	15
Have you ever consider being in the “friends with benefits” relationship?	Yes	36	28	28	27	49	28
	No	14	22	15	29	11	11
Have you ever been in a “friends with benefits” relationship?	Yes	**22**	**15**	18	15	32	25
	No	**28**	**35**	26	41	28	15
Have you ever considered “one-night stand”?	Yes	**30**	**22**	**25**	**19**	40	26
	No	**20**	**28**	**19**	**38**	21	13
Have you ever had sex with more than one person at the same time?	Yes	5	1	4	1	8	2
	No	45	49	40	55	53	38
Have you ever consider having sex with more than one person at the same time?	Yes	25	25	18	25	36	26
	No	25	25	26	32	25	13
Would you like to have sexual relationship with someone at least 10 years older than you?	Yes	15	13	13	14	19	11
	No	35	37	31	42	42	28
Have you ever been sexually attracted to someone at least 10 years older than you?	Yes	35	30	27	32	47	28
	No	15	20	16	25	13	11
Do you consider yourself as a person with sapiosexual tendencies?	Yes	18	20	18	27	42	30
	No	32	30	26	29	19	9
Would you like to have sexual relationship with a person with high intelligence quotient?	Yes	46	46	41	52	55	36
	No	4	4	2	5	6	4
Have you ever had sexual fantasies in public places?	Yes	28	25	16	25	45	26
	No	22	25	27	32	15	13
Do you use dating apps (i.e. Tinder)?	Yes	7	9	6	8	53	28
	No	43	41	38	48	8	11
Have you ever used dating apps (i.e. Tinder)?	Yes	23	26	21	29	34	19
	No	27	24	22	27	26	21
Was sex a taboo subject at your home?	Yes	25	27	26	32	36	21
	No	25	23	18	25	25	19
Have you ever been a victim of domestic abuse (physical violence)?	Yes	5	1	5	2	6	0
	No	45	49	39	54	55	40
Have you ever been a victim of sexual abuse. sexual harassment. or pedophilia?	Yes	1	5	2	7	0	2
	No	49	45	41	49	60	38
Have you ever been a victim of domestic abuse (psychological violence)?	Yes	10	10	9	14	11	4
	No	40	40	34	42	49	36
Do you present false information about yourself while using dating apps (i.e. Tinder)?	Yes	1	1	0	1	2	0
	No	39	37	35	42	13	11
	N/A	10	12	8	13	45	28
Have you ever presented false information about yourself while using dating apps (i.e. Tinder)?	Yes	3	1	2	0	4	2
	No	28	24	22	27	21	19
	N/A	20	25	19	29	36	19

PAE is measured on the dominant hand. The significant differences have been bolded in red. Data are presented as %.

**Table 6B:** Self-created questionnaires regarding sexuality.

Question	Answer	PAE		PAE			
				Female		Male	
		high	low	high	low	high	low
How old were you when masturbated for the first time?	less than 14	32	25	19	22	53	30
	14-16 years old	7	12	7	15	8	6
	16-18 years old	5	5	8	6	0	4
	Over 18	3	2	5	4	0	0
	I have never masturbated	3	6	5	9	0	0
How old were you when you had physical intercourse for the first time. which did not ended up with sex?	less than 14	9	7	6	7	15	6
	14-16 years old	13	15	7	14	23	17
	16-18 years old	17	15	18	16	15	13
	Over 18	7	8	8	13	4	0
	I have never had such contacts	4	5	5	6	4	4
How old were you when you had sex for the first time?	less than 14	1	0	0	0	2	0
	14-16 years old	4	4	1	5	9	2
	16-18 years old	17	14	13	13	23	17
	Over 18	17	19	19	24	15	11
	I have never had sex	11	13	11	15	11	9
How often do you masturbate?	Multiple times a day	1	1	0	0	2	4
	Once a day	4	1	0	1	9	2
	3-4 times a week	12	7	5	4	25	13
	Twice a week	9	10	5	11	15	9
	Once a week	7	4	7	5	6	4
	Less than once a week	18	25	27	36	4	8
How often do you have sexual fantasies before falling asleep?	Everyday	6	1	2	1	11	2
	3-4 times a week	9	10	9	12	9	8
	Twice a week	9	7	6	5	13	9
	Once a week	9	6	7	5	11	8
	Less than once a week	17	26	19	34	15	13
Who is usually the object of your sexual fantasies? You can choose more than one answer. 1	Stranger	3	1	2	1	4	0
	Current boyfriend/girlfriend	28	28	27	33	30	19
	Ex boyfriend/girlfriend	6	5	4	6	9	4
	Familiar person	9	12	7	12	13	11
	Not applicable	4	5	4	5	4	6
In which public places do you have sexual fantasies?	At home	12	12	12	13	13	11
	Classes at the university	7	8	6	9	9	6
	Cinema/theater	3	4	4	5	2	4
	Meeting with friends	9	4	4	2	17	6
	At work	1	0	0	0	2	0
	Not applicable	18	22	19	27	17	13
What attracts you to a sexual partner the most?	Intelligence	**9**	**12**	9	12	9	11
	Intriguing personality	**15**	**22**	18	27	11	15
	Physical attractiveness	**20**	**8**	9	6	36	11
	Self-confidence	**6**	**8**	7	12	4	2
What was the biggest age difference between you and your sexual partner?	1 year or less	14	14	14	16	13	11
	2-3 years	10	14	7	16	15	9
	4-5 years	9	6	6	7	13	4
	6-9 years	5	4	5	5	6	2
	10 years or more	3	1	4	1	2	2
	Not applicable	9	11	8	11	11	11
What was the major reason for entering into a “friends with benefits” relationship?	Fear of emotional involvement	5	3	4	5	8	0
	Desire to experiment	7	5	7	5	8	6
	Desire of sexual need fulfilling without commitments	10	4	8	4	13	6
	No partner or problem in finding a partner	1	4	0	4	4	6
	Not applicable	26	33	25	40	28	23
Have you ever had sexual risk behaviors? You can choose more than one answer.	Sex with Strangers	3	6	2	5	4	8
	Unprotected sex	10	8	9	7	11	9
	Sex under the influence of alcohol or drugs	10	7	5	8	19	6
	No. never	27	29	27	36	26	17
Why do you use dating apps (i.e. Tinder)?	Desire of finding a boyfriend/girlfriend	3	7	4	4	**2**	**11**
	Casual sex	1	0	0	0	**4**	**0**
	Verification of self-attractiveness	4	1	2	2	**6**	**0**
	Desire of finding friends	1	2	1	4	**2**	**0**
	Not applicable	41	40	36	47	**47**	**28**
Why have you used dating apps (i.e. Tinder)?	Desire of finding a boyfriend/girlfriend	9	16	12	15	**6**	**17**
	Casual sex	4	1	0	0	**9**	**4**
	Verification of self-attractiveness	4	2	2	4	**6**	**0**
	Desire of finding friends	7	6	6	9	**8**	**0**
	Creation of “alter ego”	1	1	1	1	**0**	**0**
	Not applicable	26	24	22	27	**32**	**19**
How many first Tinder dates have you gone on which ended in sex?	None of the first Tinder dates ended up with sex	13	8	**15**	**8**	9	8
	With one person	2	4	**1**	**6**	4	2
	With 2-3 people	3	4	**0**	**4**	8	6
	More than 4 people	0	2	**0**	**2**	0	2
	Not applicable	32	31	**27**	**36**	40	23

The significant differences have been bolded in red. Data are presented as %.

## 4 Discussion

According to data from the nationally representative General Social Survey, which included 26,620 participants from 1989 to 2014, American adults engaged in sexual activity approximately nine fewer times per year in the early 2010s compared to the late 1990s (21). Engaging in sexual activity has been linked to various physical and psychological health benefits (22). Furthermore, research indicates that happiness levels among adults over 30 have decreased since 2000 (23). Additionally, engaging in more frequent sexual activity is linked to increased well-being and greater happiness (24). Both men and women who maintain an active sex life experience improved physical fitness, enhanced happiness, better cognitive function, and increased life expectancy (24–26). There is a body of evidence that has shown inadequate sleep, sleep disturbances, and sleep disorders significantly impact various facets of human health, including sexual function (27). In our study, we pose a question: is there a correlation between mental disorders, sexual dysfunction, and PAE, as indicated by DR?

### 4.1 Relationship between PAE, sexuality and mental disorders

Recent research suggests correlations between PAE, often measured via the 2D:4D ratio, sexuality, and mental health traits. Studies have found that lower 2D:4D ratios, which indicate higher androgen exposure, are associated with traits like spatial abilities and, in some cases, sexual orientation. Sadr et al. (28) found that 2D:4D ratios in the right hand were more consistently associated with gender dysphoria (GD) than those in the left hand. The meta-analysis also highlighted smaller effect sizes for the left hand compared to the right hand, emphasizing the asymmetry between the two. Our study did not reveal any correlations between the anthropometric measurements taken on the dominant hand (2D, 4D, 2D:4D) with questionnaires regarding sexuality and mental disorders.

Research into PAE suggests that variations in early hormone levels can significantly impact mental health outcomes, with associations noted in conditions such as depression, anxiety, and gender dysphoria. For example, research on mental health has found that individuals with lower 2D:4D ratios may have a greater predisposition to certain behaviors linked to addictive tendencies or even mood disorders. As we studied literature, eighty-four females aged 18 to 40 years were assessed using the Female Sexual Function Index (FSFI), the Hospital Anxiety and Depression Scale (HADS), and the Type-D Personality Scale (DS14), which encompasses negative affectivity and social inhibition subscales. Additionally, subscales from the Eating Disorder Inventory-3 (EDI-3), specifically Drive for Thinness, Bulimia, and Body Dissatisfaction, were employed. Specifically, findings show that individuals with lower 2D:4D ratios may exhibit non-heterosexual orientations more often than those with higher ratios. This link supports theories of prenatal hormonal influences on sexual orientation. In our study, sexual function was assessed through the ASEX to gain insights into key areas of sexual activity, including sexual desire, arousal, erectile function, orgasmic ease, and satisfaction. To deepen the understanding of sexual health, SSQ across domains of Closeness, Petting, and Sex, along with the SapioQ and SSI, offering a comprehensive view of individual sexual function, were used. To capture changes in mental health, questionnaires regarding depression (PHQ-9), mood (MDQ), anxiety (GAD-7), general health (SF-12), and sleep (PSQI) were included. Unfortunately, in our study, no significant correlations were observed between PAE and questionnaires regarding sexuality and mental disorders. In the Mann-Whitney test analysis, women tend to have higher ASEX and SapioQ scores than men but lower SSQ, SF-12 (MCS) and SF-12 scores. Our analysis did not reveal significant correlations within the male samples. This aligns with prior research, which has consistently reported smaller effect sizes in studies examining the relationship between 2D:4D ratios and various traits in males compared to females. The potential link between a low 2D:4D ratio and traits like aggression, higher risk-taking, or impulsivity is thought to play a role in mental health vulnerability. For example, research on mental health has found that individuals with lower 2D:4D ratios may have a greater predisposition to certain behaviors linked to addictive tendencies or even mood disorders. The study by Buchholz et al. explored the relationship between markers of prenatal androgen exposure (PAE) and sexual behaviors in young men. It found significant correlations between PAE markers, such as the 2D:4D ratio, and online sexual compulsivity and erectile function. The study suggests that individuals with lower 2D:4D ratios, which are indicative of higher prenatal testosterone exposure, may be more prone to behaviors linked to sexual compulsivity. These findings align with previous research linking PAE to impulsive behaviors and risk-taking tendencies (29).

### 4.2 Relationship between sexuality and mental disorders

Mental disorders significantly impact patients’ quality of life with varying degrees of severity. In 2022, Leichsenring et al. published an umbrella review and meta-analytic evaluation of the efficacy of psychotherapies and pharmacotherapies for mental disorders in adults. A total of 102 meta-analyses were included, comprising 3,782 RCTs and 650,514 patients. These covered a range of conditions, including depression, anxiety, post-traumatic stress, etc. After over fifty years of research, thousands of RCTs, and significant financial investment, the effect sizes of psychotherapies and pharmacotherapies for mental disorders remain modest, suggesting a potential ceiling effect in current treatment approaches. To drive meaningful progress, a paradigm shift in research methodologies may be necessary (30). Mental disorders are often associated with sexual disorders, which further diminish the quality of life for affected individuals (21).

Sexual function disturbances can be easily correlated to depression. A reduction in the frequency of sexual activity can result in a mood decline and the onset of other psychological disorders (21). PAE (measured on the dominant hand) does not differentiate any of the questionnaires used in the study, also when considering gender (**Table 2**). Women have significantly higher ASEX and SapioQ scores than men but lower SSQ (Sex), SF-12 (MCS) and SF-12 scores. Consequently, questionnaires regarding sexuality were correlated with those assessing mental disorders (**Table 4**). In our study group, SSI was positively correlated with questionnaires regarding mental disorders (PHQ-9, GAD-7, MDQ) and negatively correlated with SF-12 and its subscales. In women, SSI was positively correlated with PHQ-9 and GAD-7 in both high and low participants, but MDQ was only correlated in the high PAE group. In our study group, ASEX was negatively correlated with SF-12(MCS), but the correlations are weak. In men, SSI was also positively correlated in high PAE males with PHQ-9, GAD-7, and MDQ (**Table 4**). The observed correlations in this study provide insights into the relationship between mental disorders, libido, and sexual satisfaction in young adults, particularly within a population largely composed of medical students. This may have influenced the inability to identify a correlation given the high demands and stress associated with medical training, this group may experience fluctuations in libido and sexual satisfaction due to external stressors. Stress, intense academic commitment, and limited personal time are known to impact sexual desire and satisfaction levels, potentially moderating the effects of PAE on sexual health outcomes in young adults. In our findings, females reported higher scores on sexual satisfaction indices compared to males, which may reflect differences in how men and women in this age and academic group perceive and prioritize sexual satisfaction amidst their studies. These results suggest that while PAE does not provide any insight into sexual satisfaction, libido in young adults appears to be significantly influenced by lifestyle and psychosocial factors intrinsic to high-stress academic environments. This interaction might explain why correlations between PAE, libido, and sexual satisfaction, though present, remain weak. Further research should explore how academic stress and lifestyle changes during early adulthood might either mask or amplify the biological influences of PAE on sexual health, as well as the gender-specific nature of these effects.

### 4.3 Relationship between PAE and sexual orientation

No differences in sexual orientation were observed in participants when taking into account PAE and Gender (**Table 5**). The study included participants with a mean age of 22.28 ± 1.99 years, primarily recruited at the university. This may have influenced the inability to identify a correlation between DR and sexual orientation. However, a review of the current literature shows that Leinung and Wu conducted a study in 2017 to investigate the correlation between DR and gender identification in the transgender New York clinic population (31). In a population of 118 transgender individuals undergoing hormone therapy, there were 50 FtM and 68 MtF for a 2D:4D ratio. Cisgender volunteers were chosen for the control group measurements. It was found that FtM individuals had a smaller dominant hand DR (0.983 ± 0.027) when linked with cisgender female controls (0.998 ± 0.021, p = 0.029). However, the ratio was similar to that of the control males. A distinctly different group of participants was recruited in comparison to our study. On the other hand, Richards et al. (32) conducted a study on self-measured 2D:4D ratio and gender variance, including 71 adults divided into four groups of participants: cisgender females, AFAB (assigned female at birth and includes female-to-male participants as well as those with other gender variant identities), cisgender males, and AMAB individuals (assigned male at birth and includes male-to-female participants as well as those with other gender variant identities). The study found that right-hand 2D:4D ratios were lower in cisgender males compared to the AMAB group and marginally lower in cisgender females relative to the AFAB group. Additionally, their study revealed a significant effect on the left-hand 2D:4D ratio. Heterosexuals’ left-hand 2D:4D ratio was lower than individuals who chose to specify gender as ‘other’ or ‘prefer not to say.’ However, no significant changes were observed with homosexuals. The limitations of this study include the small sample size and also observations made by Voracek et al. (33), suggesting that the reliability of self-measured DR may differ in accuracy from those taken by experienced researchers.

Additionally, Turan et al. investigated the 2D:4D ratio as a potential indicator of PAE in individuals with gender dysphoria (GD), comparing these individuals with same-sex siblings and cisgender controls. Individuals assigned female at birth transitioning to male and 22 individuals assigned male at birth transitioning to female (46 FtM, 22 MtF) and matched controls, FtM individuals displayed significantly masculinized 2D:4D ratios in the right hand compared to female controls, although no significant differences were observed between siblings or male controls. No feminization was observed in the MtF group’s 2D:4D ratios, suggesting that PAE may play a role in the FtM profiles but not MtF GD profiles. Our study did not include transgender participants, resulting in a relatively homogeneous sample that limited our ability to make similar observations.

As was mentioned in the Introduction section, Siegmann et al. (10) proposed an interesting finding from a meta-analysis of existing literature. A meta-analysis, including 17 samples and over 3,600 participants, showed a significant difference in 2D:4D between male-to-female (MtF) transgender individuals and male controls. These findings suggest that variations in 2D:4D, particularly in MtF individuals, reflect the influence of PAE on gender identity, adding evidence to the role of early hormone environment in shaping sexual and gender preferences. The study found a significantly higher (feminized) left-hand 2D:4D ratio in MtF transgender individuals compared to male controls. Both the original study and the meta-analysis found no significant differences in 2D:4D ratios between female-to-male (FtM) transgender individuals and female controls, indicating that prenatal androgen exposure may not play a similar role in FtM gender identity development (10).

Manning et al. (34) used data from a BBC Internet Study comprising 209,317 participants to examine whether prenatal sex steroids influence transgender identity via the 2D:4D ratio. The results indicated that natal males identifying as female (M→F) and male-to-female (MtF) individuals exhibited higher and more feminized 2D:4D ratios than natal males who identified as male (M→M), suggesting a reduction in prenatal testosterone exposure in M→F and MtF individuals. Furthermore, no significant differences in 2D:4D were observed between individuals assigned with female-to-male (F→M) to FtM individuals compared to natal females, suggesting a role of prenatal sex steroids in shaping gender identity in F→M of FtM individuals. A meta-analysis on 2D:4D ratios as a potential marker for PAE examined sexual orientation across 18 male and 16 female independent samples, totaling over 5,800 participants. Findings confirmed that heterosexual women displayed higher (more feminine) 2D:4D ratios than lesbians for both hands, while no significant differences were observed between heterosexual and gay men. Ethnicity emerged as a moderating factor in male samples, suggesting a complex interaction between early androgen exposure, sexual orientation, and ethnic background, particularly in men (35). Evidence indicates partial support for the hypothesis that PAE is associated with adult sexual orientation. This relationship appears to depend on the timing of androgen fluctuations, individual variability in androgen receptor characteristics, and potential nonlinear effects, where increased androgen may masculinize up to a threshold, beyond which feminization may occur. Additionally, androgen effects likely interact with other prenatal hormones and biochemical factors (36).

### 4.4 Relationship between PAE and sexual behaviors

In our study, we used self-created questionnaires regarding sexuality (**Tables 6A, B**). These questionaries included questions: Have you ever had a ‘one night stand’’? Have you ever considered being in a “friends with benefits” relationship? We show that higher PAE participants were more inclined toward casual relationships, with 22% reporting prior “friends with benefits” relationships compared to lower PAE counterparts (15%), particularly among females. These results suggest a greater openness to this type of relationship among those with higher PAE. Similarly, when considering the idea of a “one-night stand,” 30% of participants with high PAE reported having considered it, while only 22% of those with low PAE expressed similar thoughts. The same trend was observed in females but not males (**Table 6A**). No differences were observed when considering the gender (**Table 6B**). Participants with lower PAE more often preferred partners with intelligence and intriguing personalities over physical attributes, regardless of gender. However, Manning’s research group has been studying the correlation of DR with many diseases, inhibitions, or inclinations for many years. They were the first to note that 2D:4D has a relationship with sexual behaviors. In 2000, they proposed that right-hand DR in males is negatively correlated with family size across a number of ethnic groups (37). In 2001, research showed that high sex drive and a correlated number of sexual partners might shed some light on the association between low 2D:4D and family size in men (38). In 2006, they hypothesized a negative correlation between right-hand 2D:4D ratios and the number of sexual partners (NSP) per individual. The study found that the relationship between right-hand 2D:4D NSP was independent of free testosterone, although free testosterone had a weak positive association with NSP. In a sample of 79 heterosexual and 95 homosexual Austrian men, a significant negative association between right-hand 2D:4D and NSP was observed among heterosexual men but not homosexual men, and this association remained even when controlling for age, education, occupation, and relationship status. These findings suggest that prenatal testosterone may have a lasting impact on NSP, particularly in heterosexual men. On the other hand, Pearce et al. (2018) explore the genetic influences on 2D:4D ratios and their associations with sociosexual traits, analyzing 474 participants for single-nucleotide polymorphisms (SNPs) in nine neurochemical receptor genes. Significant associations were found between 2D:4D and genetic variations in the AR, OPRM1, and AVPR1A genes. Mediation analysis suggests that in women, variations in AR and OPRM1 are linked to 2D:4D ratios, which are further associated with increased impulsivity and, in the case of OPRM1, lower perceived romantic relationship quality. This study provides preliminary evidence that genetic factors may indirectly influence sociosexual behavior through their impact on 2D:4D ratios (39).

### 4.5 Limitations

The study primarily involved young, healthy adults, which may restrict the applicability of the findings to other age groups or those with existing health issues. Although several variables were considered in the study, other possible confounding factors—such as stress, dietary habits, and environmental impacts—were not analyzed, which might influence sleep quality. The cross-sectional design of the study limits our ability to make causal conclusions. Longitudinal studies would be essential for establishing the temporal links between PAE and sleep outcomes. Moreover, the sample size for objective sleep measures was relatively limited, so future investigations with larger participant groups would strengthen our conclusions. Additionally, using self-reported data for certain measures could lead to bias, despite our attempts to confirm these findings with objective data. Data collection was also affected by the COVID-19 pandemic, potentially introducing variability in study conditions and influencing overall participation rates.

## 5 Conclusions

In conclusion, this study offers insights into the relationship between prenatal androgen exposure (PAE), mental health, sexual functioning, and social attitudes toward sexuality. The analysis revealed that women exhibited higher ASEX and SSI scores than their male counterparts, with no significant gender differences observed in other domains. This indicates the nuanced effects of sex and gender on sexual satisfaction and mental health. PAE did not significantly distinguish overall questionnaire scores. Importantly, participants with higher PAE reported increased openness to casual relationships, such as “friends with benefits” and “one-night stands,” especially among women. This suggests a potential association between elevated prenatal androgen exposure and a broader acceptance of certain sexual and social relationship patterns. These findings highlight the necessity for ongoing research into the complex role of PAE in sexual and psychosocial development and suggest possible gender-specific pathways through which it impacts mental health and sexual attitudes.

## Data Availability

The raw data supporting the conclusions of this article will be made available by the authors, without undue reservation.
